# Mirogabalin as a novel calcium channel α_2_δ ligand for the treatment of neuropathic pain: a review of clinical update

**DOI:** 10.3389/fphar.2024.1491570

**Published:** 2024-11-22

**Authors:** Fei Yang, Yan Wang, Mingjie Zhang, Shengyuan Yu

**Affiliations:** Department of Neurology, The First Medical Center, Chinese PLA General Hospital, Beijing, China

**Keywords:** central neuropathic pain, mechanism of action, mirogabalin, peripheral neuropathic pain, pharmacokinetics, voltage-gated Ca^2+^ channel

## Abstract

Neuropathic pain (NP) is often caused by diabetic neuropathy, chemotherapy, or spinal cord lesions and is associated with significant economic burden and poor quality of life. Sophisticated etiology and pathology recognized different pharmacologic interventions, and hitherto, the reported analgesic efficacy and safety of guideline-recommended drugs are not satisfactory. Overall, this article reviews the mechanism of α_2_δ ligand, the clinical pharmacokinetics, efficacy, safety and cost-effectiveness of mirogabalin for the treatment of NP, offering clinical perspectives into potential benefits of NP-related syndrome or comorbidities. Mirogabalin, a novel voltage-gated Ca^2+^ channel (VGCC) α_2_δ ligand with selective binding affinities to α_2_δ-1 than α_2_δ-2 subunit, exhibited a wider safety margin and a relatively lower incidence of adverse events compared with other gabapentinoids. Randomized-controlled trials and open-label studies have demonstrated the efficacy and long-term safety of mirogabalin in Asian patients with diabetic peripheral neuropathic pain (DPNP), postherpetic neuralgia (PHN), and central NP. Analgesic effects of mirogabalin for the single or add-on treatment on chemotherapy-induced peripheral neuropathy and orthopedic disease/postoperation-related NP were also evidenced. To date, mirogabalin is approved for the general indication of NP in Japan, PNP in South Korea, and DPNP in the Chinese Mainland and DPNP, PHN in Taiwan (China). In summary, mirogabalin emerges as a promising option for NP; further research is warranted to refine wider treatment strategies, flexible dosing in real-world setting.

## 1 Introduction

Neuropathic pain (NP) is a condition characterized by lesions or diseases in the nervous system and may lead to loss of function, persist continuously or manifest as recurrent episodes (Scholz et al., 2019). NP can be classified as peripheral neuropathic pain (PNP), which is induced by herpes zoster (HZ), diabetes mellitus (DM), cauda equina compression, radiculopathy, chemotherapy, and central neuropathic pain (CNP), associated with spinal cord injury (SCI), poststroke pain, and multiple sclerosis. NP is a major contributor to the global disease burden and has a worldwide prevalence of 6.9%–10% in the general population (Van Hecke et al., 2014). Globally, there were approximately 206 million people with DM in 2021, of which China has 140 million (IDF Diabetes Atlas, 2021). Among Chinese patients with type 2 diabetes mellitus (T2DM), the prevalence of diabetic peripheral neuropathy (DPN) was as high as 57.2% (Li et al., 2023a). The overall incidence of HZ was 5.80 per 1,000 person-years in the United States and 6.64 per 1,000 person-years in China; about 7.3%–12.8% of patients with HZ had postherpetic neuralgia (PHN) (Sun et al., 2021; Thompson et al., 2021). The sophisticated etiology and pathology recognized different mechanisms and pain phenotypes for each diagnosis, as well as the difficulty of individualized treatment algorithms under particular physiology (Finnerup et al., 2021). In China, although there is a considerable amount of population meeting the criteria for the use of analgesics, clinical management of NP is relatively inadequate because of economic burden and other reasons (Lian et al., 2022).

The European Federation of Neurological Societies (EFNS), Canadian Pain Society, and Neuropathic Pain Special Interest Group (NeuPSIG) recommended tricyclic antidepressants (TCAs), serotonin norepinephrine reuptake inhibitors (SNRIs), and gabapentinoids including gamma-aminobutyric acid (GABA) analogs as the first line of treatment for various NP conditions (Attal et al., 2010; Dworkin et al., 2013; Moulin et al., 2014). However, available first-line pharmacological interventions have been reported to exert moderately effective or insufficient responses and the required numbers to treat (NNT) was 6–7 to achieve symptomatic pain relief of 50% (Dworkin et al., 2013; Finnerup et al., 2015). In Chinese patients with diabetic peripheral neuropathic pain (DPNP), gabapentinoids including gabapentin and pregabalin were recommended as the first-line treatment, and the most common analgesics. However, indications to use pregabalin for the management of DPNP have not yet been approved for marketing in China (Chen et al., 2023). Only 28% of patients reportedly believed that the analgesics had significantly reduced their pain, whereas 42% of patients believed that the side effects were affecting their daily life routine (Lian et al., 2022). Hence, there was a substantial and unmet need for effective and safe treatment for patients with NP, which has led researchers to develop new pharmacological therapies.

**FIGURE 1:**
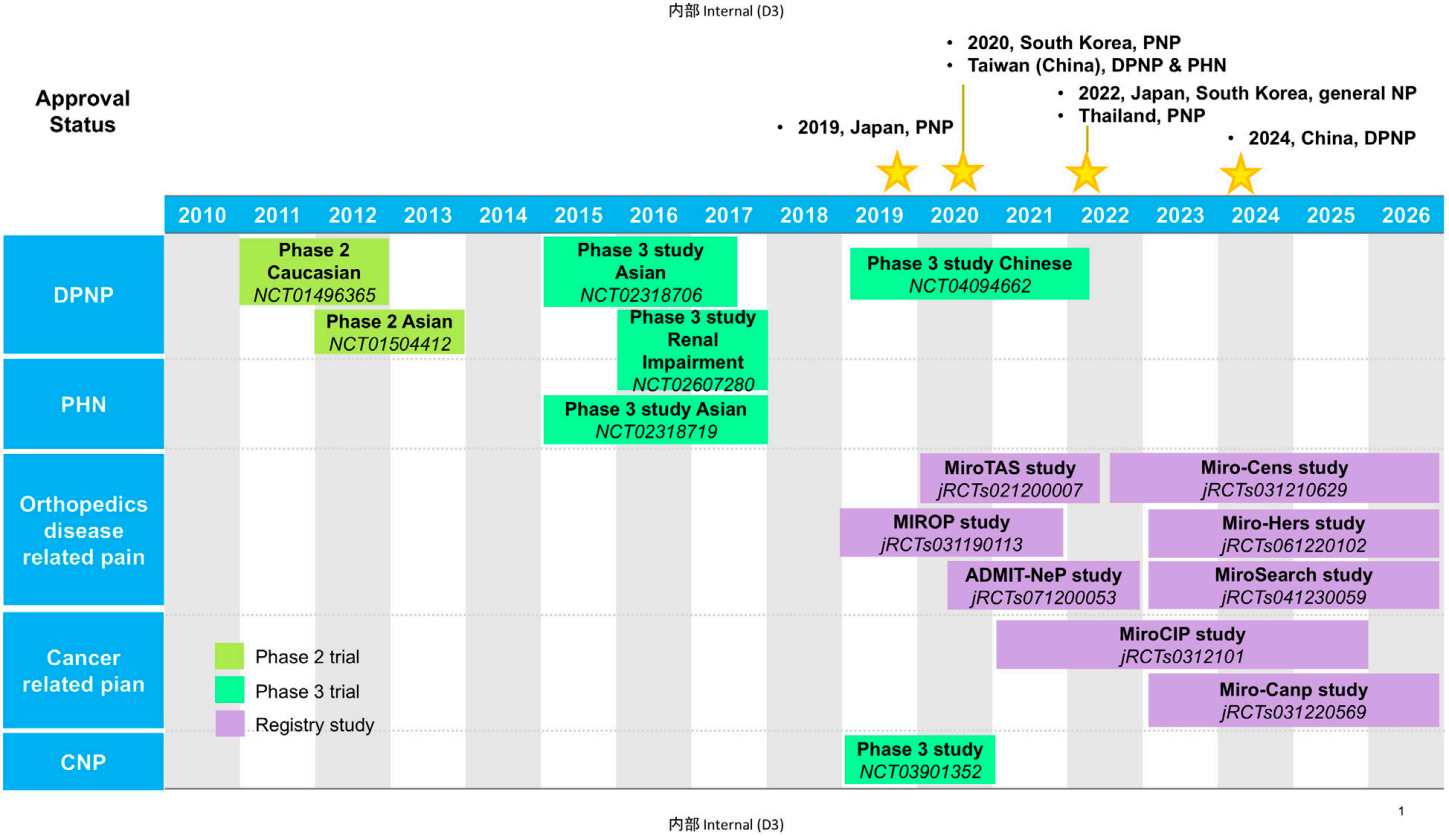
Clinical study milestones and approval status of Mirogabalin.

Mirogabalin besylate (herein called mirogabalin) is a novel ligand for α_2_δ subunit of voltage-gated Ca^2+^ channel (VGCC) developed by Daiichi Sankyo. Mirogabalin as a third member of gabapentinoids was first approved in Japan in January 2019 for the treatment of PNP and as general indication for NP including CNP in 2022. It was approved in South Korea for PNP in 2020, and then NP in 2022. In 2020, mirogabalin also got its approval for DPNP and PHN in Taiwan (China). In 2022, mirogabalin was approved for PNP in Thailand. In June 2024, based on the active observations from a phase 3 study on Chinese patients with DPNP, the Center for Drug Evaluation (CDE) of the National Medical Products Administration (NMPA) approved mirogabalin for the indication of DPNP in China (Center for Drug Evaluation of NMPA, 2023) (Figure 1). Currently, there are plenty of extensive investigations on mirogabalin for DPNP, PHN, postoperative NP, chemotherapy-induced peripheral neuropathy (CIPN), and CNP in Asian countries. In this review, we summarized the pharmacology, clinical pharmacokinetics, efficacy, and safety of mirogabalin to bridge the knowledge gap in the clinical care of NP.

## 2 General information and drug market

**FIGURE 2:**
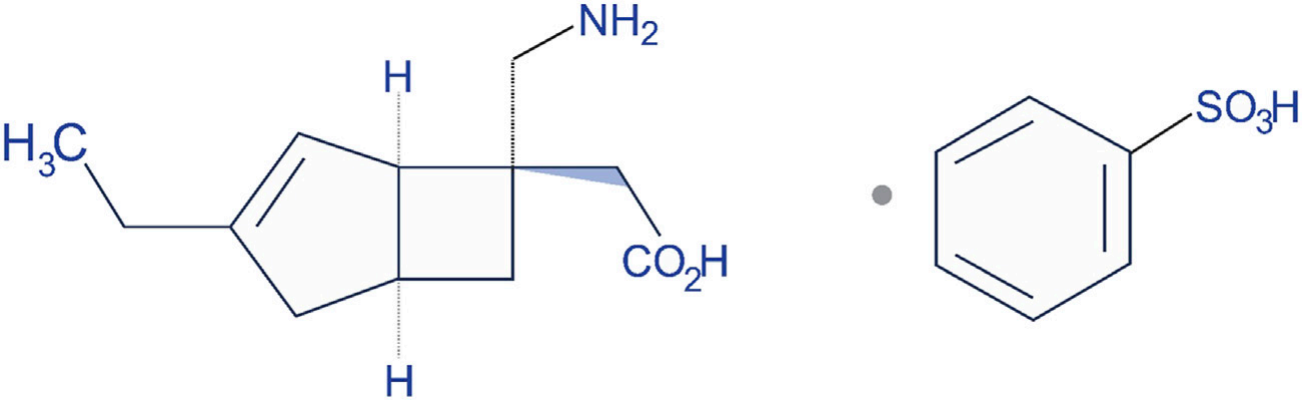
Chemical structure of mirogabalin.

Mirogabalin was launched in the market with the brand name of Tarlige^®^. The chemical name of mirogabalin is [(1*R*,*5S*,*6S*)-6-(Aminomethyl)-3-ethylbicyclo [3.2.0]hept-3-en-6-yl]acetic acid monobenzenesulfonate (Figure 2). It is packaged in 2.5, 5, 10, and 15 mg tablets. The recommended initial dose for adult patients is 5 mg twice daily (BID) with at least a weekly increase of 5 mg per dose, up to a maximum dose of 15 mg BID (TABLETS, 2023). Based on creatinine clearance (CrCl) levels as exposure to the drug increases with worsening renal function, the dosage and administration interval of mirogabalin needs to be adjusted in those patients recommended 15 mg BID for mild renal impairment, 7.5 mg BID for moderate impairment, and 7.5 mg once daily (QD) for severe impairment (Baba et al., 2020c; Kimura et al., 2021).

Mirogabalin has been approved in Japan for the treatment of NP based on the effectiveness and safety in phase 3 study including Asian patients with DPNP or PHN, CNP, and subsequently in Korea and Taiwan (Deeks, 2019; TABLETS, 2023). Consistent with international guidelines and consensuses, the use of pregabalin, gabapentin, duloxetine, and other antidepressants or anticonvulsants was recommended in patients with DPNP, PHN, CIPN, multiple sclerosis–related pain, and central poststroke pain by Chinese Medical Association. In China, pregabalin and duloxetine has no approved indication for DPNP, as patients could claim using them to relieve pain according to the Food and Drug Administration (FDA) label. The indication of DPNP got its approval by NMPA in China in June 2024. Moreover, the European Patent Office and United States Patent and Trademark Office have issued patents for mirogabalin in September 2013 and May 2011, respectively (European Patent Ofce, 2008; United States Patent and Trademark Office, 2011).

## 3 Mechanism of action

### 3.1 Binding profile to the calcium channel

The mechanism of analgesia by gabapentinoids is largely related to the reduction of dorsal horn sensitivity via binding to the α_2_δ subunit of VGCC (Chincholkar, 2018). VGCCs consist of a pore-forming α_1_ subunit and auxiliary subunits, including α_2_δ subunit (α_2_δ-1 and α_2_δ-2) (Dolphin, 2016). The α_2_δ-1 subunit is expressed in skeletal, cardiac, and smooth muscles, along with many neuronal cell types and the dorsal root ganglia (DRG), and reported to be essential for behavioral sensitivity and mechanical hypersensitivity after partial sciatic nerve ligation (PSNL) in α_2_δ-1 knockout mice (Patel et al., 2013). The α_2_δ-2 subunit is mainly identified in the brain; such as the Purkinje cells in the cerebellum, medulla, hippocampus, and striatum; and α_2_δ-2 gene deletion in mice will develop ataxia, paroxysmal dyskinesia, and absence epilepsy (Brill et al., 2004; Dolphin, 2016). *In vitro*, disassociation kinetic assay performed by radioactivity detection of ^3^H-labeled compounds using stable α_2_δ expressing 293A cell line showed higher binding affinity to α_2_δ-1 and α_2_δ-2 subunits [equilibrium dissociation constant (K_d_) = 13.5 nmol/L and 22.7 nmol/L] on mirogabalin than pregabalin (K_d_ = 62.5 nmol/L and 125.0 nmol/L) (Domon et al., 2018). Although with no significant subtype selectivity from K_d_, the dissociation half-life (t_1/2_) of mirogabalin is longer for α_2_δ-1 (11.1 h) compared with α_2_δ-2 subunit (2.4 h). Hence, mirogabalin exhibits a comparatively slower dissociation from α_2_δ-1 (dissociation rate constant [K_off_] = 0.0627 h^−1^) than α_2_δ-2 (K_off_ = 0.2837 h^−1^). In contrast, pregabalin showed equal dissociation t_1/2_ from both α_2_δ-1 and α_2_δ-2 subunits (1.4 h). The selective and unique binding characteristics of mirogabalin with α_2_δ-1 and α_2_δ-2 and its slower dissociation rate are considered to contribute to sustained analgesic effects and potential wider safety margin for the CNS side effects when compared with pregabalin that dissociates rapidly from α_2_δ subunits (Domon et al., 2018).

**FIGURE 3:**
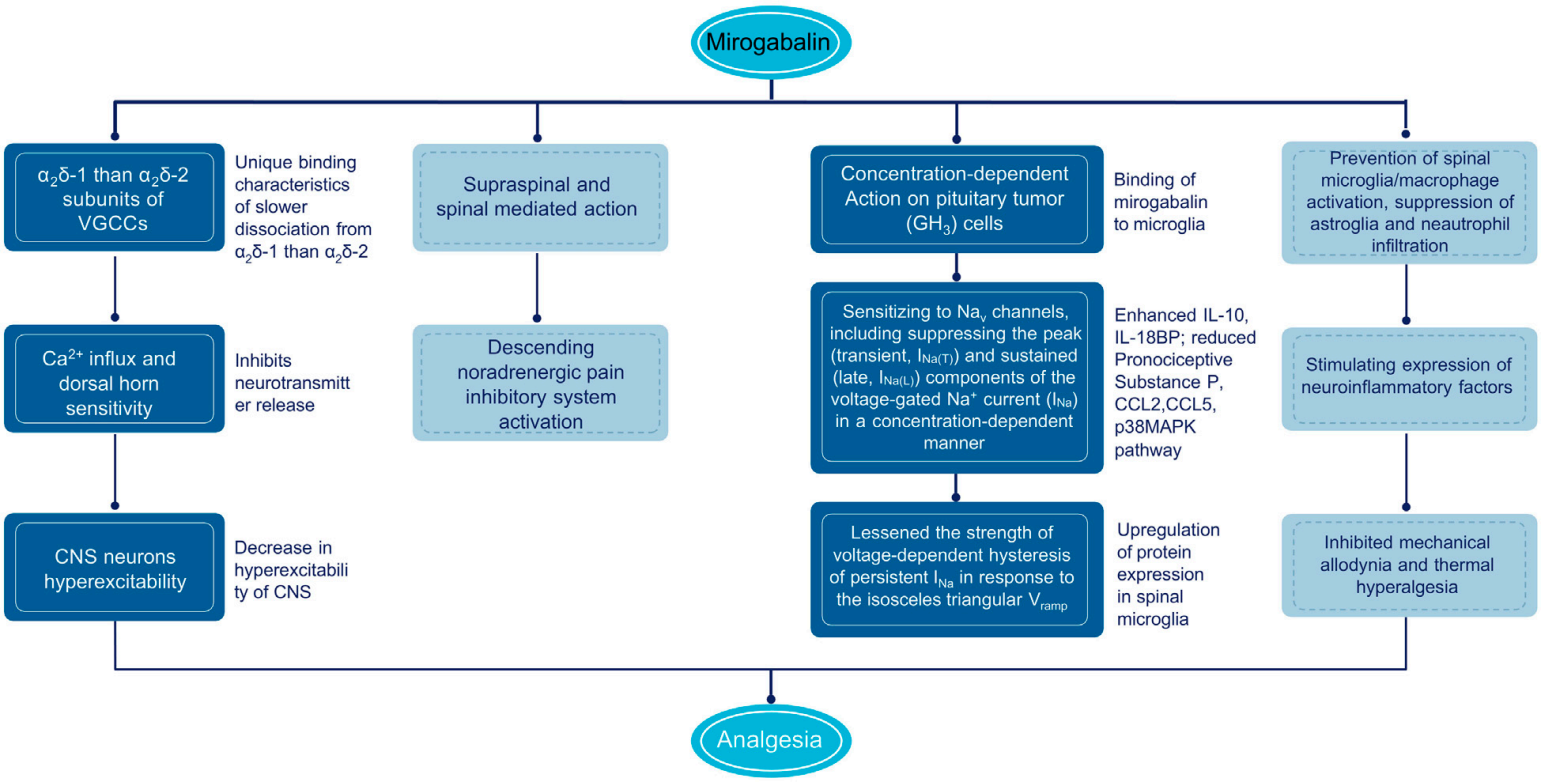
Mechanism of action of mirogabalin. CNS, central nervous system; IL, interleukin; TNF, tumor necrosis factor; VGCC, voltage gated calcium channels.

In in vitro cultured rat DRG neurons, mirogabalin (50 μmol/L), and pregabalin (200 μmol/L) inhibited the N-type calcium channel currents (Kitano et al., 2019). Similar to gabapentin and pregabalin, mirogabalin exerts analgesic effects through a multitude of actions, including inhibiting trafficking of α_2_δ-1 from the dorsal root ganglion, recycling from endosomal compartments, thrombospondin-mediated processes, and stimulating glutamate uptake by excitatory amino acid transporters (Chincholkar, 2018). In a study conducted by Oyama et al. (2021), mirogabalin has shown both supraspinal and spinal actions on thermal and mechanical hypersensitivity to ameliorate NP after partial sciatic nerve ligation. The supraspinal analgesic effects of mirogabalin involves recruitment of the descending noradrenergic pain inhibitory system by spinal activation of α_2_-adrenergic receptors. In R217A mutant mice, which substitute arginine with alanine at position 217 to significantly reduce the binding affinity to α_2_δ-1 protein, mirogabalin lost its supraspinal analgesic effects, indicating that binding to α_2_δ-1 subunit drives the efficacy of pain relief (Field et al., 2006). Furthermore, mirogabalin was also found to effectively inhibit the transient (I_Na(T)_) and late (I_Na(L)_) components of the voltage-gated Na^+^ current (I_Na_) in a concentration-dependent way in pituitary tumor (GH_3_) cells, indicating the action on excitable membranes also noticeably conferred the susceptibility to perturbations of Na_V_ channels (Figure 3) (Wu et al., 2022).

### 3.2 Inhibition of inflammation and neurotransmitters

Another possible mechanism of pain relief by mirogabalin may be indirectly caused by anti-inflammatory effects and stimulation of downstream signal inhibition. Currently, pain is considered a neuroimmune disorder, and microglia activation releases interleukin (IL)-6, IL-10, IL-4, IL-1β, tumor necrosis factor (TNF)-α, and brain-derived neurotrophic factor (BDNF), resulting in spinal dorsal horn neurons sensitization (Kawasaki et al., 2008; Wu et al., 2018). In rat model of spinal nerve ligation (SNL), intrathecal injection of gabapentin, pregabalin, and mirogabalin dose-dependently inhibited mechanical allodynia and thermal hyperalgesia (ED_50_: 30.3, 6.2, and 1.5 µg) (Ahmad et al., 2021). The mRNA and protein expression of IL-10 and β-endorphin were upregulated in both SNL rats and primary spinal microglial, illustrating the alleviation of NP by gabapentinoids through stimulating expression of spinal microglial neuroinflammatory factors. It was also reported by Zajaczkowska that in the chronic constriction injury (CCI) mice model, after intraperitoneal (i.p.) administrations of mirogabalin, a decrease in tactile and thermal hypersensitivity and enhanced mRNA of IL-10 and IL-18BP and reduced pronociceptive substance P in the spinal cord were observed (Zajączkowska et al., 2022). In addition, the antinociceptive effects of morphine, buprenorphine, oxycodone, and ketamine were potentiated when administered with mirogabalin in CCI mice, indicating the promising clinical use of mirogabalin based on opioid and ketamine analgesia of NP. Recently, Zajaczkowska added a new mechanism for the anti-inflammatory effects of mirogabalin (i.p.), revealing the prevention of spinal microglia/macrophage activation and suppression of astroglia and neutrophil infiltration, with reducing levels of pronociceptive chemokines CCL2 and CCL5, and downstream p38MAPK pathway in CCI model (Zajączkowska et al., 2023).

### 3.3 Attenuation hyperexcitability in the spinal cord

The analgesic effects of mirogabalin were assessed in a rat model of SCI at the T6/7 level with a microvascular clip for CNP. The results showed that single oral administration of mirogabalin (2.5, 5, or 10 mg/kg) significantly increased the paw withdrawal threshold with long-lasting effects (Domon et al., 2018).

## 4 Clinical pharmacokinetics

### 4.1 Pharmacokinetics in Caucasian, Asian, and Chinese

The first randomized, double-blind, placebo-controlled, phase 1 studies investigated pharmacokinetic parameters by oral administration of single (3, 5, 10, 30, 50, or 75 mg) and multiple ascending dose (5, 10, 15, and 20 mg BID or 25 mg QD to BID) of mirogabalin (Brown et al., 2018). The results revealed that mirogabalin is quickly absorbed, with a mean time to maximum plasma concentration (T_max_) of 1 h after both single and multiple ascending doses; the peak plasma concentration (C_max_ 49–1,060 and 97–426 ng/mL) and concentration-time curve (AUC_0-inf_ 184–4,896 and AUC_0-τ_ 406–1,070 ng·h/mL) increased in a dose-dependent manner, respectively. In healthy subjects well tolerated at a daily dose of ≤30 mg, the mean half-life ranged from 2.96 to 3.37 h in a single ascending dose or 3.58–4.55 h in multiple ascending doses.

**FIGURE 4:**
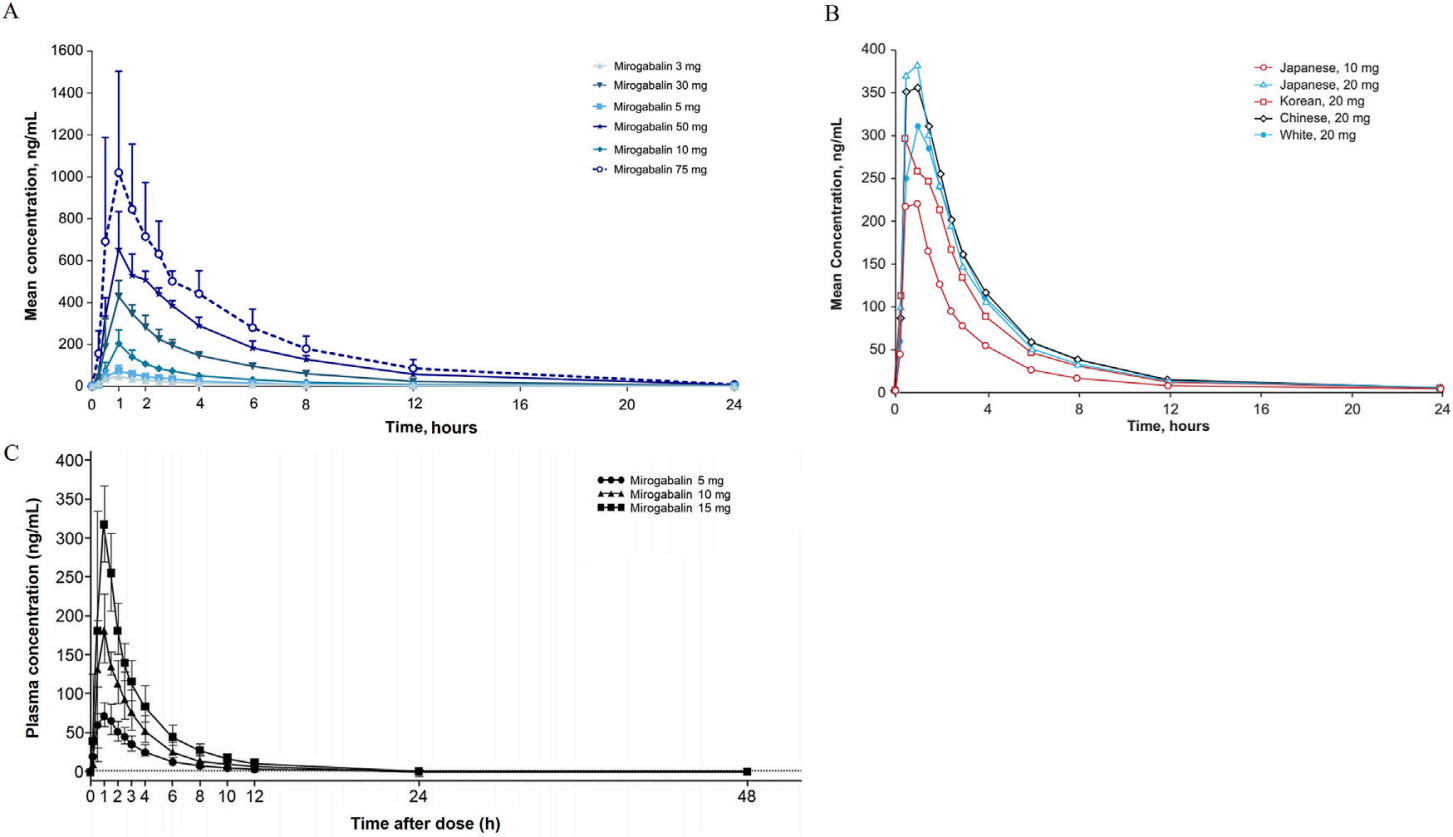
Mean concentration-time profiles after single dose administration of mirogabalin on **(A)** Caucasian, **(B)** Asian and **(C)** Chinese adults.

In a cohort of Asian subjects (Japanese, Korean, and Chinese) and an exclusively Chinese population, PK parameters of single- (5, 10, and 15 mg for the Chinese cohort; 10 and 20 mg for the Asian cohort) and multi-dose (up to 15 mg BID) mirogabalin were consistent with Caucasian participants, including T_max_ (approximately 1 h for both), t_1/2_ (2.3–9 h and 2.57–3.86 h), and CL/F (16.1–19.1 L/h and 15.9–17.6 L/h), showing no difference between ethnicities (Figure 4) (Jansen et al., 2018b; Li et al., 2023b).

### 4.2 Special considerations in renal and hepatic impairment

Mirogabalin is eliminated mainly unchanged (61%–72%) via renal excretion by filtration and active secretion, suggesting >85% oral bioavailability, whereas 13%–20% of a small fraction is metabolized by hepatic uridine 5ʹ-diphospho-glucuronosyltransferase (UGT) isoforms (Brown et al., 2018). The radioactive study of single oral administration of [^14^C] mirogabalin at a dose of 30 mg to healthy subjects showed that the main metabolites were A200-0700 (a free form of mirogabalin) *N*-glucuronide, glucuronide of oxidized A204-4455 (lactam form) (Yamamura et al., 2021).

An open-label, parallel-group study included subjects with various degrees of renal function and revealed that the CL/F of mirogabalin was decreased by 25%, 54%, and 76% in those with mild (CrCl 50–80 mL/min/1.73 m^2^), moderate (30–50 mL/min/1.73 m^2^), and severe renal impairment (<30 mL/min/1.73 m^2^); compared with 15 mg QD or BID dose with normal/mild renal impairment, similar AUC_ss_ values, but 37%–43% or 28%–32% lower C_max,ss_, were observed in subjects receiving 50% or 75% reduced dose with moderate or severe renal impairment (Yin et al., 2016). Another open-label phase 1 study confirmed that mild hepatic impairment resulted in lower concentration of A200-700 and A204-4455, and moderate hepatic impairment did not affect that of A200-700, with only a marginal decrease in plasma protein binding (approximately 22.1%), indicating mild to moderate hepatic impairment had no significant effect on mirogabalin exposure (Duchin et al., 2018). Hence, adjustment by reducing 50% or 75% of the recommended dosage is suggested in patients with moderate or severe renal impairment, but no necessity in mild renal impairment or hepatic impairment.

### 4.3 Food effect and drug-drug interaction (DDI)

Mirogabalin can be taken without food restrictions. In an open-label, crossover, phase 1 study conducted in fasted and fed healthy subjects administrated 15 mg mirogabalin, PK parameters of C_max_ were reduced approximately 18% and T_max_ delayed by 0.5 h under fed versus fasting conditions, with similar total exposure (geometric least squares mean [LSM] of AUC_0-inf_ = 94.16%; 90% CI: 91.08%–97.34%) and unaffected t_1/2_, V_z_/F, and CL/F (Brown et al., 2018).

A phase 1, open-label, crossover study assessed the DDI of inhibitor of metabolic and renal elimination on the exposure of mirogabalin as organic anion transporters 1 and 3 (OAT1/3), organic cation transporter 2 (OCT2), and multidrug and toxin extrusion (MATE) transporter (Tachibana et al., 2018). The results demonstrated that C_max_ and AUC_last_ increased by 28.7% and 76.1% when coadministered with probenecid (OAT1/3, UGT inhibitor) and 17.1% and 43.7% with cimetidine (OCT2, MATE inhibitor); renal clearance was greatly slower after coadministration of probenecid (6.67 h^−1^) and cimetidine (7.17 h^−1^) in contrast to sole mirogabalin (11.3 h^−1^), but these changes are not clinically significant. According to four randomized, double-blind, placebo-controlled studies, no clinically relevant PK interactions were observed between mirogabalin and lorazepam, zolpidem, tramadol, or ethanol after single-dose coadministration, in which only C_max_ decreased by 28% with tramadol and increased by 20% with ethanol (Jansen et al., 2018a). Concomitant administration of mirogabalin with lorazepam and ethanol increased the impairment of postural balance and attention. Therefore, its use in patients should be performed with caution, and suggestions from the physicians should be taken when mirogabalin is coadministered with lorazepam or ethanol (Tachibana et al., 2018; Yamamura et al., 2022).

## 5 Clinical efficacy

The efficacy of mirogabalin has been established in multiple studies for different types of NP. The summary of the available evidence in the treatment of PNP and CNP is presented in Table 1.

**TABLE 1:** Efficacy/effectiveness of mirogabalin clinical studies on neuropathic pain.

Author & Year	Study Design	Eligible patients	Dose of mirogabalin	Treatment period	Comparator	Endpoints	Key findings	Other results
Diabetic peripheral neuropathic pain								
Baba et al. (2019)	Phase 3, double-blind, multisite, placebo-controlled study (NCT02318706)	834 Asian patients aged ≥ 20 years with T1DM or T2DM and DPNP; painful distal symmetric polyneuropathy ≥ 6 months; VAS of the SF-MPQ ≥ 40 mm; ADPS ≥ 4	15 mg/d, 20 mg/d (10 mg BID), 30 mg/d (15 mg BID) fixed dose	Observation: 1 week; treatment (titration: 1–2 weeks; fixed-dose: 12–13 weeks); follow-up: 1 week	Placebo (2:1:1:1 randomized)	Primary: Change in ADPS from baseline at week 14 Secondary: responder rate (≥50% in ADPS), VAS in SF-MPQ, ADSIS, PGIC	LSM vs placebo of 15, 20, 30 mg/d mirogablin: −0.03, −0.15, −0.50 (*p* = 0.0027 for 30 mg OD); [LSM −1.31, −1.34, −1.47 and −1.81 for placebo and mirogabalin 15, 20 and 30 mg/d]	Significantly greater response rate for 30 mg/d (*p* = 0.0048); Significant reduction in VAS (LSM vs placebo −5.9, *p* = 0.0018) and ADSIS (−0.60, *p* = 0.0001) for 30 mg/d; Significantly more with 30 mg/d reported PGIC of ‘minimally improved or better (score≤ 3)’ (*p* = 0.0129), ‘much improved or better (score≤ 2)’ (*p* = 0.0016)
Baba et al. (2020b)	Open-label extension of phase 3 study (NCT02318706)	214 Asian patients with DPNP who completed 14 weeks of administration of mirogabalin in phase 3 study	5, 10, 15 mg BID flexible dose	Treatment (titration: 4 weeks; flexible-dosage: 48 weeks, 5 mg BID first 2 weeks, 10 mg BID second 2 weeks, 15 mg BID from week 5); follow-up: 1 week	—	SF-MPQ subscales change from baseline at week 52	The VAS [−9.8 (SD 14.06)], and other subscales of SF-MPQ generally decreased over time from baseline to week 52	—
Guo et al. (2024)	Phase 3, multicenter, randomized, double-blind, placebo-controlled trial (NCT04094662)	393 Chinese patients aged ≥18 years with T1DM or T2DM; painful distal symmetric polyneuropathy ≥6 months; VAS of the SF-MPQ ≥40 mm	15 mg BID fixed dose	Observation: 2 weeks; treatment (titration: 2 weeks, fixed dose: 12 weeks); follow-up: 1 week	Placebo (1:1 randomized)	Primary: change in ADPS from baseline at week 14 Secondary: ADPS responder rate, VAS in SF-MPQ, PGIC, ADSIS, EQ-5D-5L index and VAS	ADPS: LSM vs placebo: −0.39 (95%CI −0.74, −0.04, *p*=0.0301); [LSM −2.19 vs −1.81]	Significantly improved ADSIS: LSM vs. placebo −0.45, *p* = 0.0073), EQ-5D-5L index (LSM 0.0291, *p*=0.0107); Significantly higher PGIC of ≤ 3 (OR = 1.80, *p* = 0.0341), ≤ 2 (OR = 2.37, *p* < 0.0001)
Baba et al. (2020c)	Phase 3, multicenter, open-label study (NCT02607280)	35 Japanese patients aged ≥ 20 years with DPNP as T1DM or T2DM or PHN; CrCL15–59 mL/min; VAS of the SF-MPQ ≥ 40 mm to < 90 mm; ADPS ≥4	7.5 mg BID (CrCL30–59 mL/min), 7.5 mg QD (CrCL15–29 mL/min) fixed dose	Observation: 1 week; treatment (titration: 2 weeks, fixed dose: 12 weeks); follow-up: 1 week	—	Primary: TEAEs Secondary: change in ADPS from baseline at week 14; ADPS responder rate, SF-MPQ, ADSIS, PGIC	LSM change for total population: −1.9 (95%CI −2.8, −1.0); moderate renal impairment: −1.8 (−2.5, −1.1); severe renal impairment: −2.1 (−3.8, −0.4)	ADPS response rate ≥30%: 42.9%, ≥50%: 28.7%; For moderate and severe renal impairment, VAS in SF-MPQ: −20.8, −26.0, ADSIS: −1.4, −0.5, PGIC of ≤ 3: 76.7%, ≤ 2: 36.7%
Vinik et al. (2014) and Merante et al. (2017)	Phase 2, randomized, double-blind, placebo- and active comparator-controlled trial (NCT01496365)	452 American patients aged ≥ 18 years with T1DM or T2DM, HbA1c ≤ 10%; painful distal symmetric polyneuropathy ≥ 6 months; VAS of the SF-MPQ ≥ 40 mm; ADPS ≥4	5, 10, 15 mg/d, 20 mg/d (10 mg BID), 30 mg/d (15 mg BID) fixed dose	Screening: 3 weeks, treatment: 5 weeks (titration for mirogabalin 30 mg/d and pregabalin 300 mg/d: 1 week, fix dose: 4 weeks), follow-up: 1 week	Placebo, pregabalin 300 mg/d (150 mg BID) (2:1:1:1:1:1:1 randomized)	Primary: change in ADPS from baseline at week 5 Secondary: ADPS responder rate, PGIC, modified BPI, ADSIS	LSM for mirogablin 5, 10, 15, 20, 30 mg/d, pregabalin vs. placebo: −0.22, −0.53, −0.94, −0.88, −1.01 (*p* < 0.05 for 15, 20, 30 mg/d), −0.05; [LSM −1.9, −2.0, −2.3, −2.7, −2.6, −2.8, −1.8 for placebo, mirogabalin, pregabalin]	LSM for mirogablin vs. pregabalin: −0.17, −0.47, −0.89, −0.83, −0.96 (*p* < 0.05 for 15, 30 mg/d); ADPS response rate ≥30%: 56%–67%, ≥50%: 39%–44%; Significant reductions in ADSIS: mirogabalin 15 (*p* < 0.01), 20, and 30 mg/d vs. placebo (*p* < 0.05); Significantly greater PGIC of ≤ 2: mirogabalin 5, 10, 15, 20, and 30 mg/d vs placebo (*p* < 0.05)
Baba et al. (2020a)	Phase 2, randomized, double-blind, controlled study (NCT01504412)	450 Asian patients age ≥ 20 years; T1DM or T2DM; painful distal symmetric polyneuropathy ≥ 6 months; VAS of the SF-MPQ ≥ 40 mm; ADPS ≥4 (excluded HbA1c > 9.0%)	10 mg/d (5 mg BID), 20 mg/d (10 mg BID), 30 mg/d (15 mg BID) fixed dose	Observation:1 week; treatment: 7 weeks (titration: 1 week, fix dose: 6 weeks); follow-up: 1 week	Placebo, pregabalin 300 mg/d (150 mg BID) (1:1:1:1 randomized)	Primary: change in ADPS from baseline at week 7 Secondary: ADPS responder rates, SF-MPQ, PGIC, ADSIS	LSM placebo adjusted difference for mirogabalin 10, 20, 30 mg/d, pregabalin vs. placebo: −0.4, −0.4, −0.3, 0.0 (all *p* > 0.05); [LSM −1.5, −1.9, −1.8, −1.7, −1.4, −1.5 for placebo, mirogabalin, pregabalin]	Significant reduction in total score (LSM vs placebo −1.9, *p* = 0.0313) and VAS of SF-MPQ for 30 mg/d (LSM vs placebo −7.4, *p* = 0.0093), ADSIS (−0.9, *p* = 0.0002); Significantly greater PGIC of ≤ 3 for 10 mg/d: 13.9%, *p* = 0.0356
Postherpetic neuralgia								
Kato et al. (2019)	Phase 3, randomized, double-blind, placebo-controlled trial (NCT02318719)	765 Asian patients aged >20 years with PHN; ADPS: >4; VAS of the SF-MPQ: ≥ 40 mm	15, 20, 30 mg/d	Observation: 1 week, titration: 2 weeks, fixed dose: 12 weeks	Placebo	Primary: weekly change in ADPS at week 14, secondary: responder rate; pain on the VAS of SF-MPQ; and ADSIS	LSM vs. placebo: −0.41, −0.47, and −0.77 for mirogabalin 15, 20, and 30 mg/d groups, respectively, (*p* < 0.05 for all)	Responder rate: 35.0%, 45.4%, 45.1%, and 49.7% for placebo, mirogabalin 15, 20, and 30 mg/d groups, respectively; LSM change from baseline to week 14 in VAS of the SF-MPQ and the ADSIS was significantly greater in all mirogabalin groups compared with placebo
Kato et al. (2020)	Open-label, 52-week, extension study (NCT02318719)	239 Asian patients who completed 14 weeks of double-blind study	15, 20, 30 mg/d	Titration: 4 weeks, dose adjustment: 48 weeks, follow-up: 1 week	—	TEAEs; pain on the VAS, SF-MPQ subscales	VAS mean (SD) change from baseline: −12.4 (16.1)	—
Neuropathic pain due to orthopedic disease/ Post operation NP								
Miyazaki et al. (2024)	Multicenter, randomized, open-label, parallel-group study (jRCTs071200053)	128 patients who had undergone lung resection with peripheral NP	2.5, 5, 7.5, 10 mg BID	CrCL ≥ 60 mL/min: 5 mg BID in week 1, 10 mg BID in week 2, 10 or 15 mg BID from week 3; CrCL 30 to < 60 mL/min: 2.5 mg in week 1, 5 mg BID in week 2, 5 or 7.5 mg BID from week 3	NSAID and/or acetaminophen	Primary: Change in VAS from baseline to week 8; Secondary: S-LANSS score ≥12 at week 2, 4 and 8, PDAS, ADL EQ-5D-5L and QOL	VAS from baseline to week 8: −51.3 mm in mirogabalin vs. −47.7 in NSAID (*p* = 0.161)	S-LANSS score ≥12: 50% at baseline to 20% at week 8 (*p* = 0.003); 41.5% at baseline to 30.2% at week 8 in NSAID group (*p* = 0.134). Mirogabalin vs. NSAID - PDAS score: −24.1 ± 14.1 vs.−14.4 ± 14.8, *p* < 0.001; EQ-5D-5L score, 0.3363 ± 0.2127 vs. 0.1798 ± 0.1922, *p* < 0.001
Nikaido et al. (2022)	Randomized, open-label, parallel group, interventional study (jRCTs021200007)	220 patients with leg pain due to radicular type of LSS	5, 7.5, 10, 15 mg BID	CrCL ≥ 60 mL/min: 5 mg BID in weeks 1–2, 10 mg BID in weeks 3–4, and 15 or 10 mg BID after Week 5; CrCL 30 to < 60 mL/min, 2.5 mg BID weeks 1–2, 5 mg BID weeks 3–4, and 7.5 or 5 mg BID after week 5	NSAIDs	Primary: change in VAS leg pain score from baseline, secondary: EQ-5D-5L, PGIC	LSM change in VAS score: 24.1 mm (mirogabalin and NSAIDs) and −14.2 mm (NSAIDs), both *p* < 0.0001 vs. baseline. The difference in LSM: −9.9 [95% CI, −18.0, −1.8], *p* = 0.0174	EQ-5D-5L score in mirogabalin and NSAIDs vs. NSAIDs: mean difference, 0.0529 [0.0036, 0.1022], *p* = 0.0357. Proportions of patients with PGIC scores ≤ 3 and ≤ 2 in mirogabalin and NSAIDs vs. the NSAIDs group: 76.2% vs. 50.0%, *p* = 0.0006, and 47.6% vs. 32.4%, *p* = 0.0523
Kim et al. (2021b)	Retrospective study	52 patients treated with mirogabalin for lower extremity radiculopathy due to LSS or LDH	10 mg/d	8 weeks	—	NRS for leg symptoms and sleep disturbance, the NRS and RDQ scores for LBP, and QOL score	NRS for leg symptoms after 8 weeks: 1.8 ± 1.4 (*p* < 0.05); mean RDQ score: 2.4 ± 3.0	—
Chemotherapy-induced peripheral neuropathy								
Sugimoto et al. (2021)	Retrospective study	163 pancreatic cancer patients who underwent FOFIRINOX or GnP therapy and diagnosed with chemotherapy induced NP	5 or 10 or 15 or 20 or 30 mg/d	2, 4, 6 weeks	Pregabalin	Improvement in chemotherapy induced NP at 2, 4, or 6 weeks after treatment	Rate of pain improvement in mirogablin vs. pregabalin: 2 weeks 84.6% (11/13) vs. 33.3% (7/21), *p* = 0.005; 4 weeks, 6 weeks: 92.3% (12/13) vs. 33.3% (7/21), *p* = 0.001	—
Misawa et al. (2023)	Exploratory, interventional, open-label, single-arm study (jRCTs031210101)	58 patients experiencing moderate to severe CIPN while undergoing oxaliplatin- or taxane-containing chemotherapy for colorectal, gastric, non-small-cell lung, or breast cancer	5 and 15 mg BID	12 weeks	—	Primary: change in NRS pain score from baseline to week 12 Secondary: Changes from baseline to weeks 4 and 12 in NRS scores in the last 7 days for tingling and sleep disturbance	NRS pain score from baseline to week 12: Mean change = −1.5 [−2.3, −0.8], *p* < 0.001 NRS score for tingling Mean change = −1.2 [−1.9, −0.4], *p* = 0.003 NRS score for sleep disturbance Mean change = −0.2 in from baseline to week 12 [−0.8, 0.4], *p* = 0.534	Patients with baseline NRS of ≥ 6 experienced a 44.0% reduction in score from baseline to week 12 (LOCF): mean change:− 3.3 [− 5.0, − 1.5], *p* = 0.002
Saito et al. (2022)	Case report	Breast cancer patient with chemotherapy induced NP	5 or 10 mg BID	6 weeks	—	NRS for numbness and pain	NRS reduced from 5/10 to 3/10 for numbness and from 8/10 to 5/10 for pain	—
Central neuropathic pain								
Ushida et al. (2022)	Phase 3 randomized, double-blind, placebo-controlled study (NCT03901352)	300 patients with traumatic SCI; C4 to T12 SCI identified by MRI; stable CNP after SCI for ≥3 months before screening SFMPQ: ≥40 mm	5 mg BID for 1 week, 10 mg BID for 1 week, and 10 or 15 mg BID for 12 weeks	Observation: 1 week, titration: 2 weeks, maintenance: 12 weeks	Placebo (1:1 randomized)	Primary: change from baseline in the weekly ADPS at week 14, Secondary: ADPS responder rate, VAS, PGIC, NPSI, ADSIS, EQ-5D-5L	LSM difference vs placebo −0.71 [−1.08, −0.34], *p* = 0.0001	Responder rates [odds ratio 1.91 (1.11, 3.27) for the ≥30%; 2.52 (1.11, 5.71) for the ≥50%]. LSM difference vs placebo for SF-MPQ: −2.4 [−3.8, −1.1], ADSIS −0.71 [−1.04, −0.38], and NPSI −7.7 [−11.1, −4.4] scores
Ushida et al. (2023)	Open-label extension of phase 3 study (NCT03901352)	210 patients: 106 patients CNP-SCI complete 14-week phase 3 period, newly recruited 94 CNP-SP and 10 CNP-PD	10 or 15 mg BID	Treatment (titration: 5 mg BID first 2 weeks, 10 mg BID second 2 weeks; maintenance: 10 or 15 mg BID 47 weeks; taper: 10 or 15 mg QD 1 week); follow-up: 1 week	—	Primary: TEAEs Secondary: SF-MPQ subscales, VAS in SF-MPQ	SF-MPQ VAS mean (SD) change from baseline to week 52 CNP-SCI: − 2.3 (21.13) mm CNP-CP: − 17.0 (24.99) mm CNP-PD: − 17.1 (35.32) mm	All other SF-MPQ subscales (sensory score, affective score, total score, and present pain intensity) decreased at week 52

ADPS, average daily pain score; ADSIS, average daily sleep interference score; BID, twice daily; BPI, brief pain inventory; CNP, central neuropathic pain; CNP-SCI, central neuropathic pain from spinal cord injury; CI, confidence interval; d, day; EQ-5D-5L, EuroQol five-dimensional descriptive system; FOLFORINOX, 5-fuorouracil, oxaliplatin, irinotecan, and leucovorin; GnP, gemcitabine plus nab-paclitaxel; LBP, low back pain; LDH, lumbar disc herniation; LOCF, last observation carried forward; LSM, least square mean; LSS, lumbar spine stenosis; MRI, magnetic resonance imaging; NP, neuropathic pain; NPSI, neuropathic pain symptom inventory; NRS, numerical rating scale; PGIC, patient global impression of change; PHN, postherpetic neuralgia; QOL, quality of life; RDQ, Roland–Morris Disability Questionnaire; SCI, spinal cord injury; SF-MPQ: Short-form McGill Pain questionnaire; TEAEs, treatment-emergent adverse events.

### 5.1 Peripheral neuropathic pain

#### 5.1.1 Diabetic peripheral neuropathic pain

##### 5.1.1.1 Phase 3 placebo-controlled study involving Asian patients

In a double-blind, multisite, placebo-controlled, phase 3 study (NCT02318706) reported by Baba et al. (2019), mirogabalin has a balanced efficacy and safety in Asian patients [Japan, Korea, Taiwan (China), and Malaysia] with dose-dependent pain relief results. A total of 834 patients aged ≥20 years with type 1 diabetes mellitus (T1DM) or type 2 diabetes mellitus (T2DM) and diagnosed DPNP at least 6 months were randomized to receive mirogabalin 15 mg/d (15 mg once daily; n = 164), 20 mg/d (10 mg BID; n = 165), and 30 mg/d (15 mg BID; n = 165) including 1–2 weeks step-wise dose titration and placebo (n = 330). At week 14, the primary end point average daily pain score (ADPS) from baseline was −1.34, −1.47 and −1.81 for mirogabalin 15, 20 and 30 mg/d, respectively, and −1.31 for placebo. The LSM change in ADPS of mirogabalin over placebo was −0.03, −0.15 and −0.50, respectively, in which a dosage of 30 mg/d demonstrated statistically significant benefit (*p* = 0.0027). The decrease in APDS started from week 1 in all mirogabalin treatment groups, significantly greater for 30 mg/d compared with placebo. Mirogabalin 30 mg/d showed a significantly higher responder rate of ≥50% improvement in APDS vs placebo (*p* = 0.0048).

There was a significantly better change from baseline to week 14 of the visual analog scale (VAS) of the short-form McGill pain questionnaire (SF-MPQ) and average daily sleep interference score (ADSIS) rated by patients in mirogabalin 30 mg/d dose (*p* = 0.0018 and 0.0001). In the patient global impression of change (PGIC), >30 mg/d recorded “minimally improved or better” (score ≤3: 70.3% vs 58.8%, *p* = 0.0129) or “much improved or better” (score ≤2: 40.0% vs 26.1%, *p* = 0.0016). The results indicated improvement in the QOL with patient satisfaction.

##### 5.1.1.2 Phase 3 open-label, long-term study involving Asian patients

In an open-label, extension study of phase 3 study (NCT02318706), 214 patients from Japan, Korea, and Taiwan (China) received mirogabalin for 52 weeks (4-week titration with 5 mg BID, followed by a 48-week flexible dose of 10 or 15 mg BID) (Baba et al., 2020b). The VAS (mean change: −9.8) and the other subscales of SF-MPQ (sensory score: −1.2; affective score: −0.3; total score: −1.5; present pain intensity: −0.2) were all decreased from baseline to week 52, demonstrating long-term analgesic effects of mirogabalin in patients with DPNP.

##### 5.1.1.3 Phase 3 placebo-controlled study involving Chinese patients

Guo et al. (2024) conducted a phase 3, multicenter, randomized, double-blind study (NCT04094662) in Chinese patients aged ≥18 years with T1DM or T2DM and DPNP. A total of 393 patients were randomized to receive mirogabalin or placebo for a 2-week titration of 5 or 10 mg BID and a 12-week fixed 15 mg BID period. The change from baseline in weekly ADPS at week 14 was evaluated as the primary end point. Mirogabalin elicited significant improvement over placebo (*p* = 0.0301) with an LSM difference of −0.39 [95% CI (−0.74, −0.04), *p* = 0.0301]. The LSM change in ADPS from baseline was −2.19 for mirogabalin and −1.81 for placebo. The responder rate of ≥30% (54.1% vs 46.2%) and ≥50% (29.1% vs 26.4%) reduction in APDS from baseline to week 14 was numerically higher in the mirogabalin group in comparison with placebo, with no significance, which may be because of the high placebo response and high baseline scores in the placebo group (6.09 vs 5.60 in Asian patients) (Baba et al., 2019).

Patients receiving mirogabalin had improved VAS of SF-MPQ (LSM vs. placebo: −3.3, *p* = 0.0929). Notably, the percentage of PGIC as “minimally improved or better” (87.2% vs. 79.2%, *p* = 0.0341) and “much or very much improved” (63.8% vs. 42.6%, *p* < 0.0001) were both greater in those treated with mirogabalin than in those treated with placebo. Mirogabalin showed significant change from baseline to week 14 regarding ADSIS (LSM vs. placebo: −0.45, *p* = 0.0073), index value (0.0291, *p* = 0.0107), and VAS (2.8, *p* = 0.0457) of EuroQol 5 Dimensions 5 Levels (EQ-5D-5L). Therefore, mirogabalin was found to be safe and effective in Chinese patients with DPNP as in Asian patients from other countries/regions.

##### 5.1.1.4 Phase 2, placebo, active-controlled study

In a phase 2, randomized, double-blind, placebo, active-controlled study (NCT01496365), 913 patients from the US at the age of ≥18 years with T1DM or T2DM (HbA_1c_ ≤10%) and DPNP for ≥ 6 months were randomized to mirogabalin 5 mg/d (5 mg once daily), 10 mg/d (10 mg once daily), 15 mg/d (15 mg once daily), 20 mg/d (10 mg BID) and 30 mg/d (15 mg BID) treatment group; pregabalin 300 mg/d treatment group; or placebo group (Vinik et al., 2014). At week 5, mean changes in ADPS from baseline were −2.0, −2.3, −2.7, −2.6 and −2.8 for the mirogabalin dose ascending, −1.8 for pregabalin, and −1.9 for placebo. The LSM differences were statistically significant versus placebo for mirogabalin 15, 20, and 30 mg/d (−0.94, −0.88 and −1.01, *p* < 0.05) and versus pregabalin for mirogabalin 15 and 30 mg/d (−0.89 and −0.96, *p* < 0.05). Mirogabalin 15 and 20 mg/d showed a significantly higher percentage of ≥30% reduction in ADPS (66.7% and 60.7%) from baseline to week 5 versus both pregabalin (38.0%, *p* < 0.05) and placebo (41.7%, *p* < 0.05); more percentage of 15, 20 and 30 mg/d had significantly ≥50% reduction (39.2%, 42.9% and 43.9%) versus placebo (24.1%).

Moreover, significant reductions in ADSIS were observed in the mirogabalin 15, 20, and 30 mg/d groups (−2.97, −2.52 and −2.69) compared with placebo (−1.98, *p* < 0.05), in mirogabalin 15 mg/d versus pregabalin (−1.94, *p* < 0.05) (Merante et al., 2017). In modified brief pain inventory (BPI), the subscales of interference with daily function (−2.58 vs. −1.58), worst pain intensity (−2.96 vs. −1.93), least pain intensity (−1.95 vs. −1.19), and average pain intensity (−2.32 vs. −1.55) were improved better with mirogabalin 30 mg/d than with placebo. The significant improvement in the status of PGIC as “minimally improved or better” was observed in 5, 10 and 30 mg/d group and “much or very much improved” in all dose groups of mirogabalin than placebo (*p* < 0.05). The available evidence favors the use of high-dose mirogabalin over pregabalin to some point for the treatment of patients with DPNP.

A phase 2, double-blind, randomized, placebo-controlled study (NCT01504412) conducted in Japan, South Korea, and Taiwan (China) evaluated the effect of mirogabalin versus pregabalin for DPNP caused by ineffective treatments available at the time of the study. In this study, patients (N = 450) aged ≥20 years with T1DM or T2DM and DPNP were randomized to treat with mirogabalin 10, 20, or 30 mg/d; pregabalin 300 mg/d BID; or placebo for 7 weeks (1-week dose escalation) (Baba et al., 2020a). Although nonsignificant, LSM placebo-adjusted difference in change from baseline in ADPS at week 7 was −0.4 [−1.0, 0.2] in the 5 mg BID group, −0.4 [− 0.9, 0.2] in the 10 mg BID group, −0.3 [−0.9, 0.3] in the 15 mg BID group, and 0.0 [− 0.5, 0.5] in the pregabalin group. For secondary end points, mirogabalin 30 mg/d significantly improved the VAS [LSM: −7.4 (−13.0, −1.8), *p* = 0.0093] and total score of SF-MPQ [LSM: −1.9 (−1.3, −0.4), *p* = 0.0002] and ADSIS [LSM: −0.9 (−1.3, −0.4), *p* = 0.0002].

A meta-analysis study on the efficacy of mirogabalin treatment included three randomized controlled trials (RCTs) with 1,732 patients with DPNP (Alyoubi et al., 2021). Mirogabalin showed a significant superior reduction in ADPS for 3, 4, and 5 weeks and observed a significant increase in the patient’s proportion with ≥30% and ≥50% reduction in ADPS when compared with pregabalin and placebo.

#### 5.1.2 Postherpetic neuralgia

##### 5.1.2.1 Phase 3, placebo-controlled study

The efficacy of mirogabalin in patients with PHN has been established in phase 3 studies (Kato et al., 2019; Kato et al., 2020). In 2019, Kato et al. (2019) reported a multicenter, double-blind, placebo-controlled phase 3 study (NCT02318719) involving Asian patients with PHN and assessed the efficacy of mirogabalin based on the change from baseline with ADPS. A total of 765 patients were randomized to receive mirogabalin 15 mg/d (15 mg once daily; n = 153), 20 mg/d (10 mg BID; n = 153), 30 mg/d (15 mg BID; n = 155), or placebo (n = 304). At week 14, 15–30 mg/d doses of mirogabalin were well tolerated, and a statistically significant improvement in pain was observed with mirogabalin in ADPS LSM versus placebo: −0.41 (*p* = 0.0170), −0.47 (*p* = 0.0058), and −0.77 (*p* < 0.0001). The LSM change from baseline in ADPS was −1.61, −1.68 and −1.97 for mirogabalin dose ascending and −1.20 for placebo. The proportion of patients reporting ≥30% reduction in ADPS was also significantly higher than placebo in all three dosing regimens of mirogabalin.

Moreover, the LSM change from baseline in VAS of the SF-MPQ (−5.1, −5.7 and −7.8 for 15, 20 and 30 mg/d) and ADSIS (−0.50, −0.48 and −0.76) was significantly greater for mirogabalin than placebo. Significant improvements observed in more patients in the 15 mg/d mirogabalin group as a PGIC score of “much improved or better” (36.2% vs 26.4%, *p* = 0.0318) and in 20 and 30 mg/d mirogabalin groups as “minimally improved or better” (69.3% and 69.0% vs 54.5%, *p* = 0.0025 and 0.0028) versus placebo, suggesting a possible improvement in activities of daily living and QOL (Kato et al., 2019).

##### 5.1.2.2 Phase 3, open-label, long-term study

Furthermore, an open-label extension study of the phase 3 study (NCT02318719) established the efficacy of a long-term flexible dosing regimen of mirogabalin 10 or 15 mg BID for 52 weeks in PHN (Kato et al., 2020). A total of 239 patients who completed the week 14 period were eligible for this 52-week extension study. In terms of efficacy, improvements in SF-MPQ subscales at week 52 were observed (sensory score: −1.5; affective score: −0.3; total score: −1.8; present pain intensity: −0.3 and VAS: −12.4). The VAS score decreased gradually from baseline through the extension study and remained stable (Kato et al., 2020). Although limited, the evidence on the efficacy of mirogabalin in patients with PHN is stable in the long term, and further studies with mirogabalin for the treatment of PHN in non-Asians are warranted.

#### 5.1.3 Postoperative NP

It is anticipated that mirogabalin treatment will provide pain relief in patients with NP after surgery. Miyazaki reported a multicenter, randomized, open-label, parallel-group, interventional trial (jRCTs071200053) that assessed the effectiveness of mirogabalin in postoperative NP after thoracic surgery (Miyazaki et al., 2024). Patients diagnosed with NP after lung resection and a VAS score of ≥40 mm will receive either the monotherapy with conventional pain-relieving agents (nonsteroidal anti-inflammatory drugs [NSAIDs] and/or acetaminophen) or mirogabalin add-on (10–15 mg BID for CrCl ≥60 mL/min and 5–7.5 mg BID for CrCl ≥30 and <60 mL/min) for 8 weeks. The LSM changes of VAS [difference: −3.6 (−8.7, 1.5), *p* = 0.161], ≥30% (98.0% vs. 92.5%, *p* = 0.364) and ≥50% (94.0% vs. 92.5%, *p* = 1.000) reduction in ADPS from baseline to week 8 in mirogabalin add-on group and conventional group were similar. Notably, in patients with self-administered Leeds Assessment of Neuropathic Symptoms and Signs (S-LANSS) score ≥12, mirogabalin add-on group had significantly reduced score (from 50% to 20%, *p* = 0.003) while conventional treatment group had no significant decreasing (from 41.5% to 30.2%, *p* = 0.134). Changes in the Pain Disability Assessment Scale (PDAS) score and EQ-5D-5L also showed significance with the involvement of mirogabalin.

#### 5.1.4 NP caused by orthopedic disease

The MiroTAS is a multicenter, randomized, open-label study that evaluated the efficacy and safety of mirogabalin when given as an add-on therapy in 220 enrolled patients with lumbar spinal stenosis (LSS) taking NSAIDs (jRCTs021200007) (Nikaido et al., 2022). Patients randomly received mirogabalin plus NSAIDs (n = 110) or NSAIDs alone (n = 104) according to package inserts; mirogabalin dose was adjusted based on renal function (CrCl ≥60 mL/min: 15 or 10 mg BID; CrCl ≥30 and <60 mL/min: 7.5 or 5 mg BID). At week 12, the LSM change in VAS score from baseline was −24.1 in the combination group and −14.2 in the NSAID monotherapy group (difference: −9.9, *p* = 0.0174). A significantly greater improvement in the EQ-5D-5L score (mean difference: 0.0529, *p* = 0.0357) and a higher proportion of PGIC scores ≤3 (76.2% vs. 50.0%, *p* = 0.0006) and ≤2 (47.6% vs. 32.4%, *p* = 0.0523) were observed in those treated with mirogabalin and NSAIDs in comparison with NSAIDs.

A retrospective study has also validated the effect of mirogabalin on PNP caused by orthopedic disease (Kim et al., 2021b). This study included 60 patients who had lower extremity radiculopathy owing to LSS or lumbar disc herniation (LDH) and treated with mirogabalin (flexible dose maximum to 30 mg/d) for PNP caused by orthopedic disease were assessed to compare the pre- and post-administration for leg symptoms and sleep disturbance, the NRS and for low back pain (LBP), and the EQ-5D-5L data. After 8-week therapy, significantly better improvements in leg symptoms of numerical rating scale (NRS) 1.8 versus 6.4 in pre-treatment, low back pain (LBP) in NRS (3.2 vs. 5.2), and Roland–Morris Disability Questionnaire (RDQ, 2.4 vs. 7.5) were observed. Sleep disturbance of NRS (9.7 vs. 5.9) was also improved, and the QOL was higher as EQ-5D-5L score (0.75 vs. 0.54, all *p* < 0.05). There are several studies ongoing in Japan (jRCTs061220102, jRCTs041230059, and UMIN000037150) that investigated the efficacy of mirogabalin in patients with PNP caused by orthopedic diseases.

#### 5.1.5 Chemotherapy-induced peripheral neuropathy

Although limited evidence is available, studies have shown the effectiveness of mirogabalin in relieving CIPN (Sugimoto et al., 2021; Saito et al., 2022). An earlier study by Sugimoto et al. (2021) retrospectively analyzed the efficacy of mirogabalin and pregabalin in the treatment of CIPN. Patients with pancreatic cancer received chemotherapy regimens FOLFIRINOX (combination of 5-fluorouracil, oxaliplatin, irinotecan, and leucovorin) or GnP (gemcitabine plus nab-paclitaxel) and diagnosed CIPN during the treatment course were included in the study (n = 34; mirogabalin group: n = 13; pregabalin group: n = 21). Both mirogabalin and pregabalin reported effectiveness in improving CIPN but a significantly higher rate of improvement was observed with mirogabalin (2 weeks: 84.6% vs. 33.3%, *p* = 0.005; 4 and 6 weeks: 92.3% vs. 33.3%, *p* = 0.001). The discontinuation rate of mirogabalin was 15.4% and that of pregabalin was 52.4%. Hence, mirogabalin might be the first choice for CIPN in patients with pancreatic cancer. In a prospective, single-arm study (MiroCIP), patients with cancer prescribed with mirogabalin (5–15 mg BID) for moderate to severe CIPN while undergoing oxaliplatin- or taxane-containing chemotherapy showed a 30.9% decrease [mean change: −1.7 (−2.4, −1.0), *p* < 0.001] in the NRS pain score. Meanwhile, a 44% reduction [mean change: −3.3 (−5.0, −1.5), *p* = 0.002] in pain score from baseline to week 12 was observed in patients with a baseline NRS of ≥6, which shows the effectiveness of mirogabalin in chemotherapy-treated patients with cancer and moderate-to-severe CIPN (Misawa et al., 2023). Mirogabalin (5 mg BID increased to 22.5 mg/d) attenuated pain and numbness because of a reduction in NRS (from 8 to 5 and from 5 to 3), and duloxetine further decreased NRS value to 1 for both, suggesting the synergistic effect of this combination. In a case report of a 53-year-old patient with breast cancer, grade 2 CIPN and grade 1 symptoms were observed during adjuvant docetaxel and cyclophosphamide therapy, and both worsened when receiving eribulin (Sugimoto et al., 2021). Further evidence on the efficacy of mirogabalin for CIPN in patients with different types of tumors (UMIN000041467), primary breast cancer (jRCTs031220001), gastrointestinal cancer (UMIN000049555), or in gemcitabine plus nab-paclitaxel therapy (UMIN000038742) is worth expecting.

### 5.2 Central neuropathic pain

#### 5.2.1 Placebo-controlled study

Ushida et al. (2022) first evaluated the efficacy and safety of mirogabalin in adult patients with CNP from Japan, Korea, and Taiwan (China) in a randomized, double-blind, placebo-controlled, phase 3 study (NCT03901352). Patients aged ≥20 years who experienced traumatic SCI for ≥6 months and stable CNP for ≥3 months, with VAS ≥40 mm, were enrolled. A total of 300 patients were randomized 1:1 to receive mirogabalin or placebo for 14 weeks (titrated dose for 2 weeks and fixed dose for 12 weeks: 10 or 15 mg BID for CrCl ≥60 mL/min and 5 or 7.5 mg BID for CrCl 30–60 mL/min). At week 14, a statistically significant improvement was observed with mirogabalin in the change from baseline in the weekly ADPS when compared with placebo [LSM difference: −0.71 (−1.08, −0.34), *p* = 0.0001]. Mirogabalin also showed higher responder rates of ≥30% [OR: 1.91 (1.11, 3.27)] and ≥50% [OR: 2.52 (1.11, 5.71)] for weekly ADPS than placebo. Moreover, more significant improvements in SF-MPQ [LSM: −2.4 (–3.8, −1.1)], ADSIS [LSM: −0.71 (–1.04, −0.38)], and neuropathic pain symptom inventory [NPSI: −7.7 (–11.1, −4.4)] scores were observed in the mirogabalin group versus the placebo group.

#### 5.2.2 Open-label, long-term study

A recently published, open-label, extension study demonstrated the long-term efficacy of mirogabalin in treating CNP in Asian patients (Ushida et al., 2023). Patients with CNP (n = 210) caused by SCI (n = 106), Parkinson’s disease (PD, n = 94), and central poststroke pain (CPSP; n = 10) received mirogabalin for 52 weeks, including 5 or 10 mg BID for 4-week titration, 10 or 15 mg BID for 47-week maintenance, and 10 or 15 mg/d for 1-week tapering. The mean VAS in SF-MPQ from baseline to week 52 was reduced gradually for patients with SCI (−2.3) and CPSP (−17.0) and rapidly at weeks 12 and 16 for those with PD (−17.1). Patients with CNP caused by CPSP or PD had a decrease in all SF-MPQ subscales, and patients with SCI who were included in the previous 14-week trial had no significant change. This indicates the sustained efficacy in long-term treatment.

## 6 Safety and tolerability

### 6.1 Safety data in clinical trials

Several phase 1 studies involving healthy volunteers have shown a tolerable safety profile of mirogabalin at doses ≤30 mg/d when administered with fed and fasting state (Brown et al., 2018). The most common adverse events leading to treatment discontinuation were somnolence and dizziness, which were dose dependent. In a randomized, sequential, ascending-dose study on single (10–40 mg) and repeated (10 and 15 mg BID) doses of mirogabalin in Korean, Chinese, and White subjects, the results showed an acceptable safety and tolerability profile and the most common treatment-emergent adverse events (TEAEs) were consistent with previous reports and known mechanism (Jansen et al., 2018b). As mirogabalin dissociates rapidly from α_2_δ subunits, it has lesser frequency of CNS adverse effects compared with other α_2_δ ligands (Kim et al., 2021a). In a meta-analysis reported by Lian including two, nine, and three RCTs for patients with DPNP treated on mirogabalin, pregabalin, and duloxetine, the percentage of total cases of somnolence were 9.7% (75/771), 10.1% (121/201), dizziness were 8.6% (66/771), 9.9% (119/1,201) with mirogabalin, pregabalin, respectively; rates of nasopharyngitis were 14.7% (73/494), 14.0% (24/171) of mirogabalin, duloxetine (Jingxuan et al., 2021).

A phase 3 study confirmed the tolerable safety profile of mirogabalin (Baba et al., 2019). Details of TEAEs are presented in Table 2. When mirogabalin was administered to patients with DPNP, the most frequent TEAEs were nasopharyngitis, somnolence, dizziness, peripheral edema, and weight gain. TEAEs leading to treatment discontinuation occurred in 13 (3.9%), 4 (2.4%), 7 (4.2%), and 16 (9.7%) patients in the placebo, mirogabalin 15, 20, and 30 mg/d groups, respectively, and were mild to moderate, mostly resolved without treatment. In the placebo and mirogabalin dose ascending groups, 11 (3.3%), 4 (2.4%), 8 (4.8%), and 11 (6.7%) patients reported serious TEAEs, which were not of particular concern. An open-label extension study revealed the long-term safety of mirogabalin (flexible to 10 or 15 mg BID) in patients with DPNP, with 13.1% of TEAEs leading to discontinuation (Baba et al., 2020b).

**TABLE 2:** Most frequent TEAEs (≥5%) with mirogabalin for the treatment of neuropathic pain.

TEAEs	DPNP, N (%)					PHN, N (%)		CIPN, N (%)		NP related to lumbar disease, N (%)		CNP, N (%)	
	Asian patients (n = 165), mirogabalin 30 mg OD*(Baba et al., 2019)	Asian patients with long-term treatment (n = 214), mirogabalin 10 or 15 BID (Baba et al., 2020b)	Chinese patients (n = 196), mirogabalin 15 mg BID	Non-Asian patients (n = 57), mirogabalin 30 mg OD (Vinik et al., 2014)	Patients with renal impairment (n = 35), mirogabalin (7.5 mg BID for moderate, 7.5 mg OD for severe impairment) (Baba et al., 2020c)	Short-term treatment (n = 155), mirogabalin 30 mg OD*(Kato et al., 2019)	Long-term treatment (n = 237), mirogabalin 10 or 15 mg BID (Kato et al., 2020)	CIPN patients (n = 52), mirogabalin 5–15 mg BID (Misawa et al., 2023)	CIPN in patients with pancreatic cancer (n = 13), mirogabalin 5, 10, 15, 20 mg OD (Sugimoto et al., 2021)	Add-on to NSAIDs (n = 110), mirogabalin 10 or 15 mg BID (Nikaido et al., 2022)	Switching from pregabalin (n = 80), mirogabalin 10–30 mg OD (Akazawa et al., 2021)	Asian patients (n = 151), mirogabalin 10 or 15 mg BID (Ushida et al., 2022)	Long-term treatment (n = 210), mirogabalin 10 or 15 mg BID (Ushida et al., 2023)
Somnolence	24 (14.5)	20 (9.3)	12 (6.1)	9 (15.8)	4 (11.4)	37 (23.9)	36 (15.2)	7 (13.5)	—	33 (30.0)	6 (7.3)	45 (29.8)	35 (16.7)
Dizziness	18 (10.9)	16 (7.5)	13 (6.6)	6 (11.3)	2 (5.7)	24 (15.5)	26 (11.0)	5 (9.6)	1 (7.7)	28 (25.5)	4 (4.9)	13 (8.6)	16 (7.6)
Edema	—	13 (6.1)	—	—	—	11 (7.1)	14 (5.9)	—	1 (7.7)	—	—	—	24 (11.4)
Diarrhea	—	18 (8.4)	—	0 (0.0)	2 (5.7)	—	—	—	—	—	1 (1.2)	—	—
Weight gain	11 (6.7)	17 (7.9)	11 (5.6)	1 (1.8)	—	8 (5.2)	22 (9.3)	—	—	—	—	11 (7.3)	15 (7.1)
Back pain	—	11 (5.1)	—	—	—	—	9 (3.8)	—	—	—	—	—	11 (5.2)
Nasopharyngitis	27 (16.4)	58 (27.1)	—	—	8 (22.9)	20 (12.9)	39 (16.5)	—	—	—	—	12 (7.9)	23 (11.0)
Diabetic retinopathy	—	25 (11.7)	—	—	—	—	—	—	—	—	—	—	—
Peripheral Edema	14 (8.5)	24 (11.2)	10 (5.1)	2 (3.5)	3 (8.6)	—	11 (4.6)	2 (3.8)	—	6 (5.5)	—	9 (6.0)	26 (12.4)
Diabetes mellitus	—	12 (5.6)	—	—	—	—	—	—	—	—	—	—	11 (5.2)
Hypoglycemia	—	12 (5.6)	—	—	—	—	—	—	—	—	—	—	—
Constipation	—	12 (5.6)	—	3 (5.3)	—	—	11 (4.6)	—	—	—	—	9 (6.0)	13 (6.2)
Contusion	9 (5.5)	—	—	—	—	—	—	—	—	—	—	—	—
Nausea	—	—	—	1 (1.8)	2 (5.7)	—	8 (3.4)	—	—	—	—	—	—
Sensory disturbance	—	—	—	—	2 (5.7)	—	—	—	—	—	—	—	—
Hyperuricemia	—	—	25 (12.8)	—	—	—	—	—	—	—	—	—	—
UTI	—	—	21 (10.7)	0 (0.0)	—	—	—	—	—	—	—	—	—
Hyperlipidemia	—	—	21 (10.7)	—	—	—	—	—	—	—	—	—	—
Upper respiratory tract infection	—	—	13 (6.6)	—	—	—	—	—	—	—	—	—	12 (5.7)
Increase in blood creatine phosphokinase	—	—	10 (5.1)	—	—	—	—	—	—	—	—	—	—
Insomnia	—	—	—	—	—	—	8 (3.4)	—	—	—	—	—	—
Pharyngitis	—	—	—	—	—	—	8 (3.4)	—	—	—	—	—	—
Eczema	—	—	—	—	—	—	8 (3.4)	—	—	—	—	—	—
Headache	—	—	—	1 (1.8)	—	—	5 (2.1)	—	—	—	—	—	—
Hepatic function abnormal								1 (1.9)					—
Loss of consciousness								1 (1.9)					—

BID, twice daily; CNP, central neuropathic pain; DPNP, diabetic peripheral neuropathic pain; OD, once daily; NP, neuropathic pain; PHN, post-herpetic neuralgia; TEAEs, treatment-emergent adverse events; UTI, urinary tract infection.

Mirogabalin has also been shown to be tolerable in patients with PHN with no new TEAEs reported (Kato et al., 2019). At week 14, 12 patients (4.0%) in the placebo group, 8 (5.3%) in the 15 mg/d group, 16 (10.5%) in the 20 mg/d group, and 12 (7.7%) in the 30 mg/d group reported one or more TEAE resulting in discontinuation. Serious TEAEs were reported in five, two, three, and five patients in the 15, 20, 30 mg/d, and placebo groups, respectively. No serious TEAE was reported by more than one patient in any treatment group. The discontinuation rate of mirogabalin because of TEAEs was 8.4% for patients with PHN in the long-term extension study (Kato et al., 2020).

Mirogabalin, when used in combination with NSAIDs in the treatment of LSS, had higher incidence of TEAEs than NSAIDs monotherapy (60.9% vs. 14.2%) (Nikaido et al., 2022). However, the observed events were mild to moderate and did not cause any safety concerns. The most common TEAEs were somnolence (30.0%) and dizziness (25.5%), and no serious TEAEs or deaths were reported. The proportion of patients who discontinued treatment because of TEAE was 8.2% in the combined group.

In patients with pancreatic cancer, mirogabalin showed better safety than pregabalin for chemotherapy-induced NP (TEAEs leading to discontinuation: 13 vs. 21) (Sugimoto et al., 2021). Treatment interruption because of mirogabalin was reported in two patients (15.4%) with one reported dizziness and in 11 patients (52.4%) receiving pregabalin with five reported AEs. Although nonsignificant, the incidence of adverse events was lower in the mirogabalin group than in the pregabalin group (15.4% vs 33.3%).

Safety data from a double-blind phase 3 study support the use of mirogabalin in patients with CNP (Ushida et al., 2022). The proportion of patients with at least one TEAE was 78.1% in the mirogabalin group and 55.4% in the placebo group. The most common TEAEs observed with mirogabalin were somnolence, dizziness, peripheral edema, nasopharyngitis, constipation, weight gain, and mild in severity. TEAEs leading to treatment discontinuation of mirogabalin were reported in 14 patients (9.3%) and serious TEAEs occurred in 9 patients (6.0%) treated with mirogabalin.

### 6.2 Safety of mirogabalin when switching from pregabalin

A prospective, single-arm, open-label study (MIROP) assessed the safety of mirogabalin in 152 patients with PNP (34.2% orthopedic diseases, 28.3% PHN, and 0.7% DPNP) switching from pregabalin (Kimura et al., 2021). The incidence of somnolence, dizziness, and peripheral edema was reported in 41.4%, 15.8%, and 2.6% of patients, and AEs were mild in severity in most of the patients. The discontinuation rate considered because of treatment was 13.8%, and no deaths were reported. Akazawa et al. (2021) reported a multicenter, retrospective study that included patients with PNP related to orthopedic disease who switched from pregabalin to mirogabalin (n = 82) because of AEs and lack of efficacy (AE incidence with pregabalin: 100% vs 11.1%). After switching, the AE incidence of mirogabalin did not have a significant difference between the two groups (23.5% vs 15.9%). The incidence of somnolence and dizziness was 12.2% and 14.6% with pregabalin, whereas a lower rate of 7.3% for somnolence and 4.9% for dizziness was observed with mirogabalin. In a short-term retrospective study conducted involving patients with PNP, AEs such as somnolence (28.8%), dizziness (14.8%), edema (2.1%), and weight gain (0.9%) led to a switch in therapy from pregabalin to mirogabalin (Tetsunaga et al., 2020). The study showed a tolerable safety profile for mirogabalin with no new safety concerns with somnolence (26.7%), dizziness (12.3%), edema (5.9%), and weight gain (0.5%) (Tetsunaga et al., 2020).

### 6.3 Mirogabalin for patients with renal or hepatic impairment

Mirogabalin was well tolerated by Japanese subjects with normal renal function, mild to severe renal impairment, and end-stage renal disease (ERSD) but a higher incidence of TEAEs, supporting dose adjustment (Kato et al., 2018). An open-label study enrolled patients with renal impairment aged ≥20 years diagnosed with DPNP or PHN and prescribed mirogabalin 7.5 mg BID for moderate renal impairment (CrCl: 30–59 mL/min) or 7.5 mg/d for severe renal impairment (CrCl 15–29 mL/min). The overall incidence of TEAEs was 82.9% (29/35) and most of them were mild or moderate, with the most common TEAE being nasopharyngitis (22.9%) and somnolence (11.4%). TEAEs leading to discontinuation were reported by only four patients (11.4%) with moderate renal impairment (Baba et al., 2020c).

When mirogabalin was assessed in an open-label, single-dose study, only two patients with mild hepatic impairment treated with 15 mg mirogabalin daily reported mild somnolence and recovered rapidly (Duchin et al., 2018). No severe adverse events or discontinuation or death was observed in patients with mild to moderate hepatic impairment. More studies with a larger sample size on the safety of mirogabalin to determine whether dose adjustment or not for patients with hepatic impairment is warranted in the future.

## 7 Cost-effectiveness of mirogabalin

Currently, the evaluation of the cost-effectiveness of a drug is becoming an increasingly important criterion. Gray et al. (2021) analyzed the cost-effectiveness of mirogabalin 30 mg in the treatment of DPNP using a Markov model. The head-to-head base-case analysis demonstrated that mirogabalin 30 mg is a cost-effective treatment option compared with placebo with an incremental cost-effectiveness ratio (ICER) of US$15,658/quality-adjusted life years (QALY) in patients with DPNP in Taiwan (China), with an estimated QALY gain of 0.02 at an incremental cost of US$310 versus placebo. In addition, mirogabalin was cost-effective compared with pregabalin 300 mg (ICER: US$600/QALY). The cost-effectiveness of mirogabalin in Chinese patients with DPNP has also been established from the healthcare system perspective in the mainland China by a Markov model (He et al., 2023). The results showed that mirogabalin 30 mg is more cost-effective than pregabalin 300 mg, with an ICER of $6869.67/QALY [below the willingness-to-pay threshold in China ($11,339.43)].

The cost-effectiveness of mirogabalin in patients with PHN was also evaluated in Taiwan (China) (Ye et al., 2020). The cost-effectiveness of mirogabalin 30 mg with an incremental QALY gain of 0.041 was at an incremental cost of US$359 versus placebo (US$ 8786) resulting in an ICER of US$8766. Moreover, mirogabalin 30 mg was cost-effective compared with pregabalin 150 and 300 mg, with ICERs of US$16,720 and US$6535, respectively [below the WTP threshold in Taiwan (US$56,000)]. These results supported the good cost-effectiveness of using mirogabalin in Chinese patients.

## 8 Conclusion

Previously, the evidence on efficacy for the treatment for NP was insufficient with safety being a concern in the treated patients. Hence, a novel gabapentinoid, mirogabalin, which selectively binds to α_2_δ subunits with a unique mechanism of slower dissociation from α_2_δ-1 than α_2_δ-2, was developed, which emerges as a potential alternative for the treatment of NP. Several phase 3 studies have demonstrated the efficacy of mirogabalin in patients with DPNP and PHN, which ensured the clinical application in many Asian countries/regions. The post-authorization real-world studies will further evaluate the safety and effectiveness in plenty of clinical practice and may emphasize patient-tailored care for border NP. Although still in its infancy, mirogabalin has shown efficacy in palliating orthopedic disease, postoperative NP, and chemotherapy-induced NP. Furthermore, mirogabalin appears to offer superior analgesia for PNP than for CNP, and the clinical care in these patients is certainly promising. In the safety profile, patients with NP showed lower AEs after switching from pregabalin to mirogabalin, indicating a possible clinical action of switching between analgesics. In the MIROP study of patients with PNP, >70% patients were able to safely tolerate the step-wise dose titration of the effective doses of mirogabalin after switching from pregabalin to mirogabalin (Kimura et al., 2021). In patients with renal impairment, mirogabalin could be used in a dose-adjusted manner and was proved to be well tolerated, indicating its feasibility for its application on special groups, such as the elderly or patients with comorbidities, but a few studies did not show notable improvements. Hence, future research should focus on optimizing the efficacy and safety of mirogabalin in a large population for various chronic pain conditions thereby providing a wider array of therapeutic regimens for NP patients, especially in patients undergoing treatment for comorbidities. These research studies should primarily focus on examining flexible dosages in the effective range of 15–30 mg/d for mirogabalin fitting the clinical practice, with the hope of attaining better outcomes. In addition, comparative studies between mirogabalin and pregabalin, gabapentin, duloxetine, milnacipran, and amitriptyline would prove valuable in establishing the position of mirogabalin among the other analgesics for the treatment of NP.

In conclusion, the effectiveness and tolerability of mirogabalin in addressing NP signifies a valuable addition to the therapeutic options available.
